# *Haemophilus ducreyi* cutaneous ulcer contracted at Seram Island, Indonesia, presented in the Netherlands

**DOI:** 10.1371/journal.pntd.0006273

**Published:** 2018-04-12

**Authors:** Jarne M. van Hattem, Tessa J. C. Langeveld, Sylvia M. Bruisten, Marion Kolader, Martin P. Grobusch, Henry J. C. de Vries, Godelieve J. de Bree

**Affiliations:** 1 Department of Medical Microbiology, Academic Medical Center, University of Amsterdam, Amsterdam, the Netherlands; 2 Center of Tropical Medicine and Travel Medicine, Academic Medical Center, University of Amsterdam, Amsterdam, the Netherlands; 3 Department of Internal Medicine, Leiden University Medical Center, Leiden University, Leiden, the Netherlands; 4 Public Health Laboratory (PHL), Department of Infectious Diseases, GGD/Public Health Service Amsterdam, Amsterdam, the Netherlands; 5 Amsterdam Infection & Immunity Institute (AI&II), Amsterdam, the Netherlands; 6 STI Outpatient Clinic, Department of Infectious Diseases, GGD/Public Health Service Amsterdam, Amsterdam, the Netherlands; 7 Department of Dermatology, Academic Medical Center, University of Amsterdam, Amsterdam, the Netherlands; 8 Amsterdam Institute for Global Health and Development, Academic Medical Center, University of Amsterdam, Amsterdam, the Netherlands; Universidade Nova de Lisboa Instituto de Higiene e Medicina Tropical, PORTUGAL

## Abstract

**Overview:**

We describe the first case of a cutaneous ulcer caused by *Haemophilus ducreyi* imported from Indonesia to the Netherlands. Skin infections caused by *H*. *ducreyi* are uncommon in travellers and have been described in just a few case reports and were all contracted on the Pacific Islands.

**The case:**

A 22-year-old healthy male visited the Center of Tropical Medicine and Travel Medicine in February 2017 with a cutaneous ulcer of the right lateral malleolus 4 weeks after returning from Indonesia (Seram and Ambon Islands). He had noticed a small skin abrasion on the right ankle after slipping on a rock during a jungle trip on Seram Island. Back in the Netherlands, a painful ulcer developed at the same body location, and despite treatment with flucloxacillin, his complaints worsened. A swab that was taken for culture showed growth of small grey colonies that were characterised as *H*. *ducreyi* with matrix-assisted laser desorption/ionisation time-of-flight (MALDI-TOF) mass spectrometry. Treatment with ciprofloxacin for the diagnosis of *H*. *ducreyi* cutaneous ulcer was started, and the ulcer clearly diminished, leaving only a small healing ulcer.

**Discussion:**

*H*. *ducreyi* is normally the causative agent of genital ulcers but is increasingly recognised as a cause of chronic skin ulcers, e.g., in Papua New Guinea. In our patient, the infection was very likely contracted in the Maluku province of Indonesia and imported into the Netherlands. No reports of infection with *H*. *ducreyi* from Indonesia could be found in literature, but this case indicates that *H*. *ducreyi* is present in at least one of the northeastern islands of Indonesia, which is important for local healthcare. Additionally, it illustrates the role of this agent as a cause of cutaneous ulcers in previously healthy travellers.

## The case

**Fig 1:**
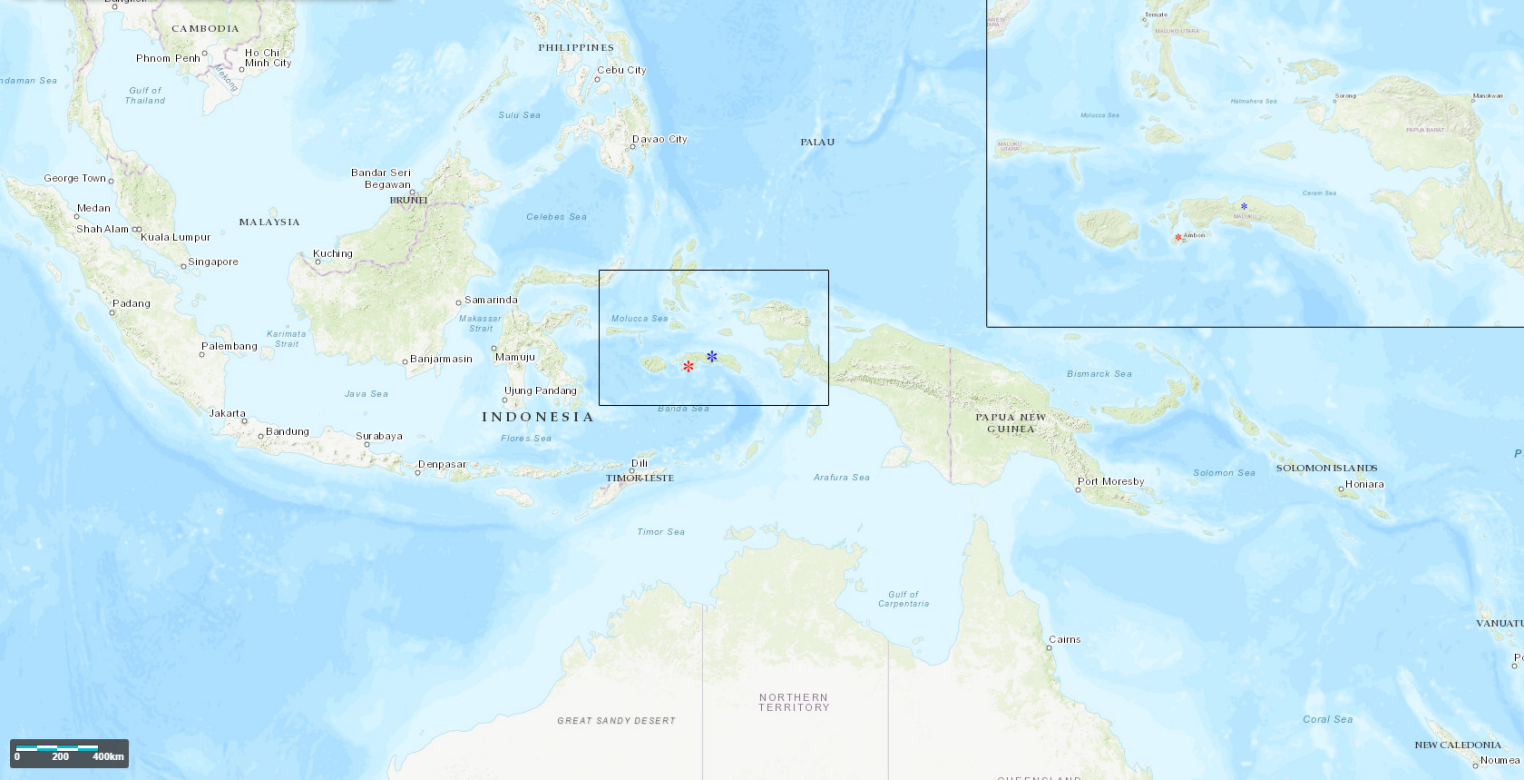
Map showing the location of Indonesian islands visited by our patient. The black box is shown enlarged in the box in the right upper corner. Seram and Ambon, islands that were visited by our patient, are indicated with a blue and red star, respectively. This map was created using the online LandsatLook Viewer at https://landsatlook.usgs.gov/.

**Fig 2:**
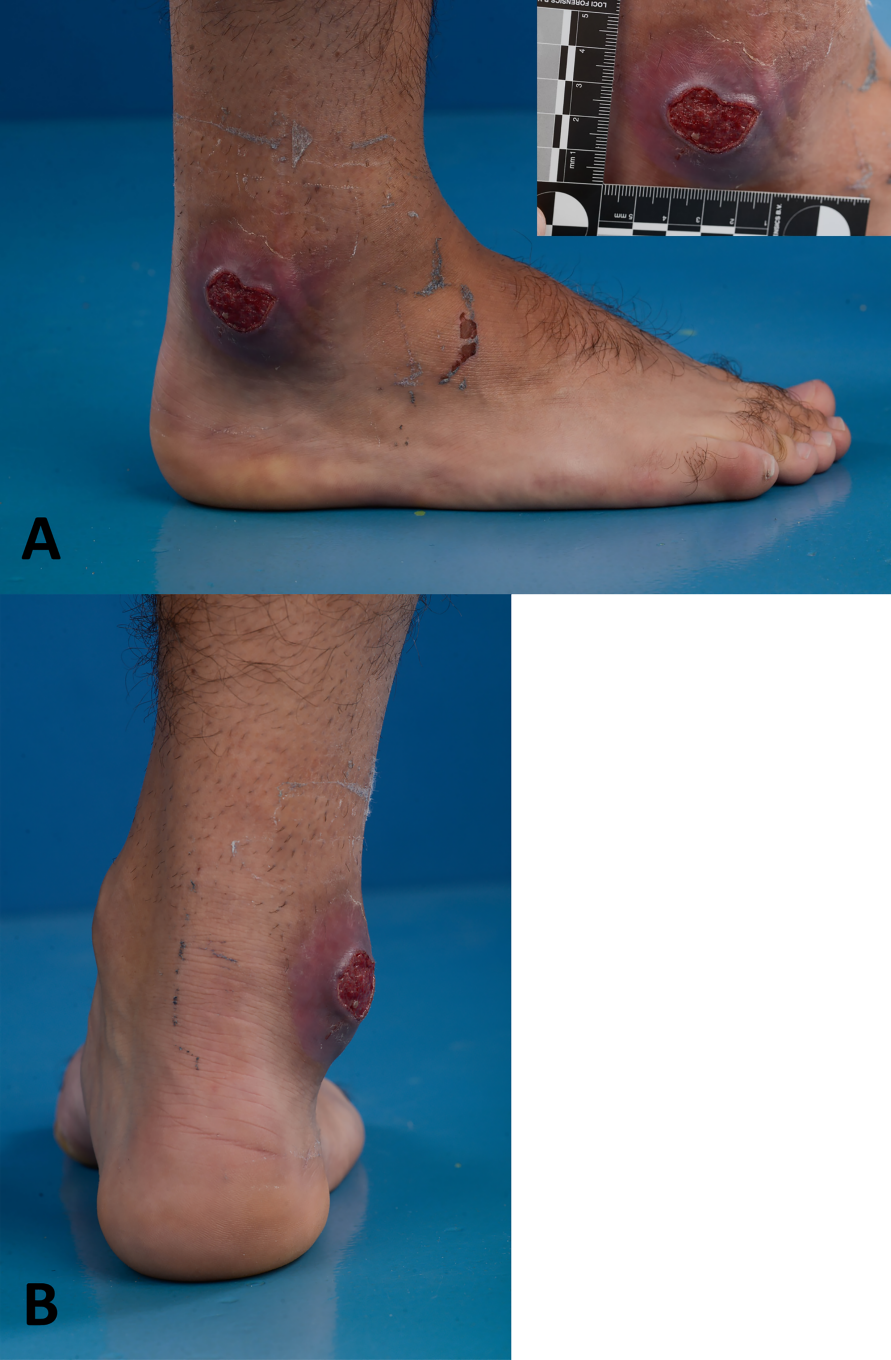
Photo of the ulcer on the right lateral malleolus showing an indurated ulcer of 3 × 3 cm with a hypergranulomatous surface, perilesional erythema and oedema, and undermined wound margins. Panel A: lateral view. Panel B: dorsal view.

A 22-year-old healthy male visited the Center of Tropical Medicine and Travel Medicine in February 2017 with a cutaneous ulcer on the right lateral malleolus. Four weeks before presentation, he returned from Indonesia (Seram and Ambon Islands, Fig 1), where he spent 3 weeks visiting friends and relatives. In the last week of his holidays, he noticed a small skin abrasion on the right ankle after slipping on a rock during a jungle trip on Seram Island. Back in the Netherlands, a painful ulcer developed at the same body location, and he visited his general practitioner who prescribed flucloxacillin 500 mg qid for 10 days for the suspected diagnosis of cellulitis. Nevertheless, his complaints worsened, and he was referred to the Center of Tropical Medicine and Travel Medicine of the Academic Medical Center (AMC) in Amsterdam. Upon physical examination, he appeared healthy with a normal blood pressure and body temperature. On the right lateral malleolus, we saw an indurated ulcer of 3 × 3 cm with a hypergranulomatous surface, perilesional erythema and oedema, and undermined wound margins (Fig 2). The ulcer was very painful upon manipulation. There was no evidence of regional adenopathy. All laboratory results were normal, including a white blood cell count of 8.4 × 10^9^/L (normal 4 − 10.5 × 10^9^/L).

**Fig 3:**
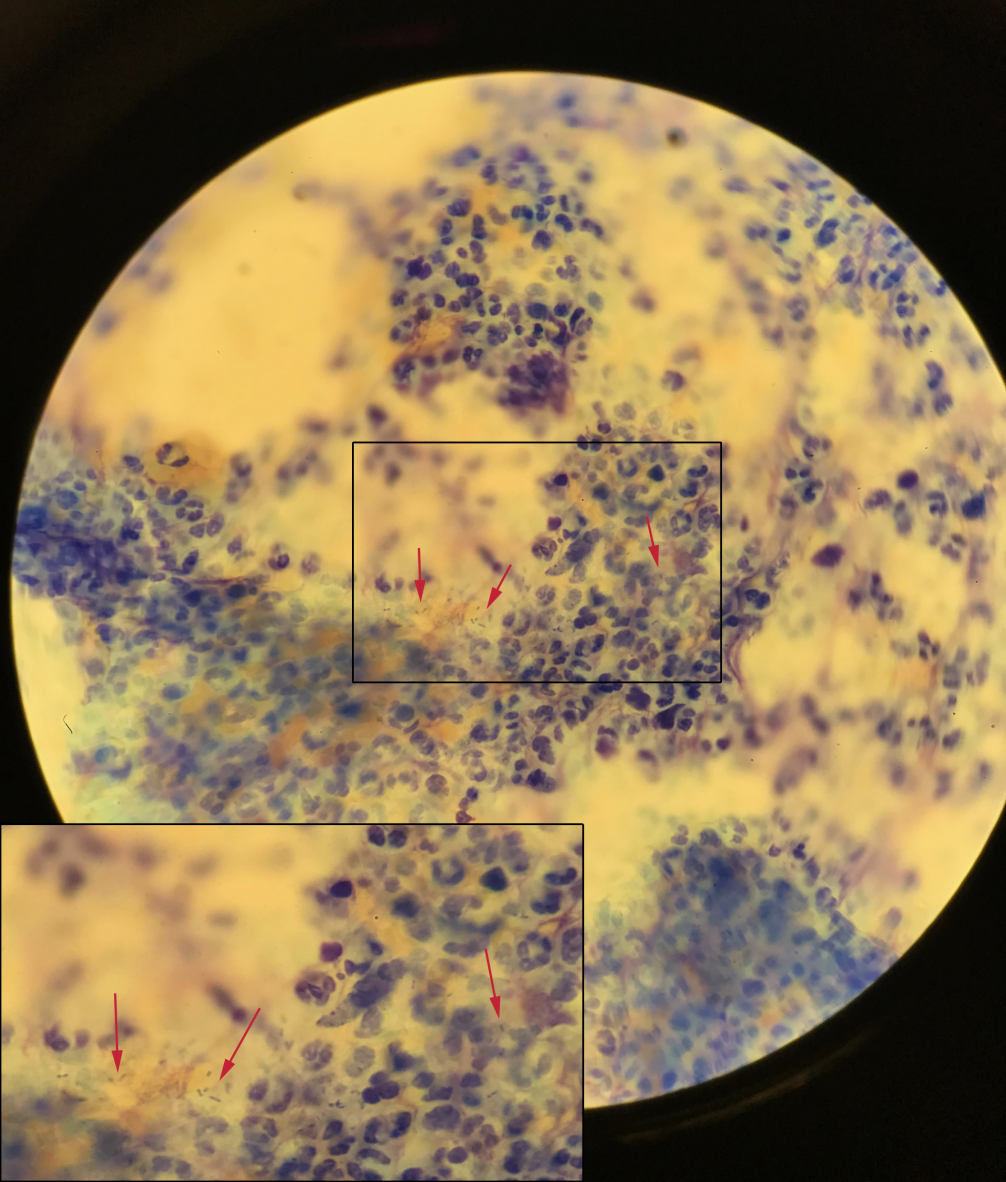
Photo of microscopy of the ulcer’s exudate smear, using Diff-Quik staining, from the wound margin taken at initial presentation showing small rod-shaped bacteria as indicated by arrows (magnification 1,000×). The box in the middle is shown enlarged in the box in the left lower corner.

**Fig 4:**
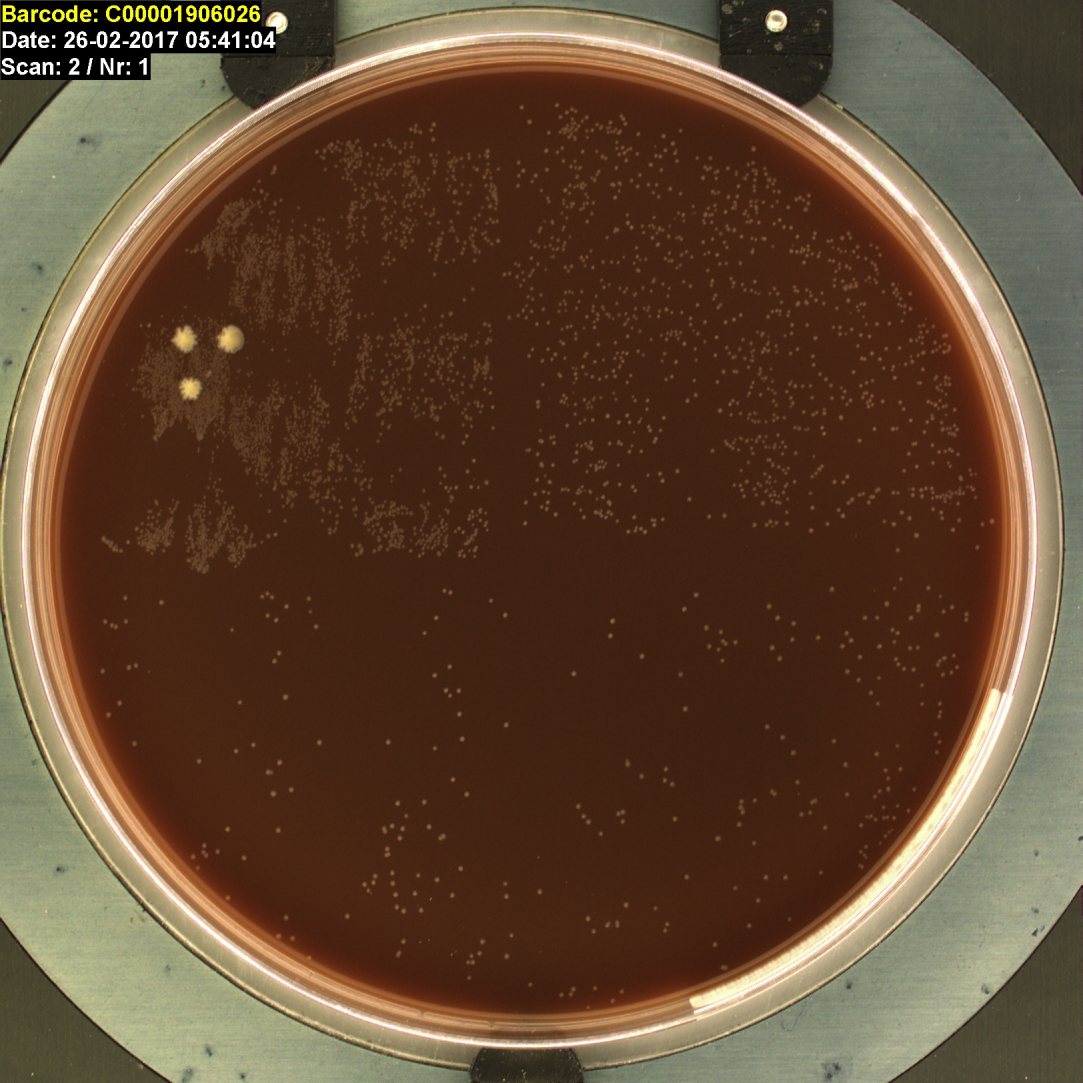
Growth of small grey colonies identified as *Haemophilus ducreyi* as seen on the chocolate agar plate after an incubation period of 48 hours. The 3 white colonies were not further specified.

A swab was taken and sent for culture, including an exudate smear from the wound margin for Diff-Quik staining. In the smear, we saw small rod-shaped bacteria (Fig 3). Culture of the wound was performed according to standard laboratory procedures. After 48 hours of incubation (5% CO_2_ at 37°C), growth of small grey colonies was observed on the chocolate agar plate (PolyViteX, bioMérieux Marcy l'Etoile, France) (Fig 4).

Colonies were characterised as *H*. *ducreyi* with matrix-assisted laser desorption/ionisation time-of-flight (MALDI-TOF) mass spectrometry (Bruker Microflex LT, Bruker, London, UK) with log(score) value of 2.067 indicating a high-confidence identification. Identification of *H*. *ducreyi* was further confirmed by specific PCR targeting 16SrRNA of *H*. *ducreyi* (performed at the Public Health laboratory [PHL], GGD Amsterdam) and generic 16SrRNA PCR (performed at the AMC) on the isolate. Sequencing of both amplified 16SrRNA gene fragments showed 100% concordance with published *H*. *ducreyi* sequences [1, 2] and sequences of a *H*. *ducreyi* reference strain that was cultured 20 years ago at the PHL.

Using the European Committee on Antimicrobial Susceptibility Testing (EUCAST) breakpoints for *H*. *influenzae*—in the absence of clinical breakpoints for *H*. *ducreyi*—susceptibility testing by disc diffusion (BD BBL Sensi-Disc, Becton Dickinson, the Netherlands) indicated that the isolate was resistant to amoxicillin and susceptible to amoxicillin/clavulanic acid, tetracycline, and cefotaxime with a positive beta-lactamase test (chromogenic cephalosporin nitrocefin; Oxoid, Wesel, Germany). Minimum inhibitory concentration (MIC) of cotrimoxazole, ciprofloxacin, and azithromycin determined by E-test (bioMérieux Marcy l'Etoile, France) were 0.08, 0.002, and <0.016 mg/l, respectively, indicating that these could all be suitable for treatment. Before treatment was started, skin biopsies from the margins of the ulcer were taken at a second visit, 12 days after the original visit to rule out atypical mycobacterial infection—since the patient had been exposed to seawater after the trauma—and endemic treponematosis caused by *Treponema pallidum* subspecies *pertenue*.

From the biopsy tissue, a few colonies of *Corynebacterium diphtheriae* biovar mitis were cultured. Cutaneous diphtheria usually presents as nonhealing skin ulcers, too. However, because of its presence in low quantity, the fact that it was toxin-negative, as determined by PCR, and the existence of an alternative diagnosis, the cultured *C*. *diphtheriae* was considered as colonisation—although a causal relationship cannot be fully excluded.

No growth of *H*. *ducreyi* was seen, and specific *H*. *ducreyi* PCR was negative for this sample. *T*. *pallidum* PCR targeting all *T*. *pallidum* subspecies as well as *Mycobacterium tuberculosis* complex PCR, mycobacterial culture from the skin biopsy, and screening for syphilis by chemiluminescence immunoassay (Liaison XL, DiaSorin, Saluggia Italy) on serum turned out to be negative, excluding (atypical) mycobacterial infection and endemic treponematosis as a cause.

After the biopsy was taken, we treated the patient with ciprofloxacin 500 mg bid for the duration of 2 weeks for the diagnosis of *H*. *ducreyi* cutaneous ulcer (HD-CU). Thereafter, his pain was completely resolved, and the ulcer clearly diminished, leaving only a small healing ulcer.

## Discussion

We describe the first case of a cutaneous ulcer caused by *H*. *ducreyi* imported from Indonesia to the Netherlands. *H*. *ducreyi* is a gram-negative coccobacillus typically causing a sexually transmitted infection (STI) called ‘chancroid’ or ‘ulcus molle’, characterised by one or more soft and painful genital ulcers and regional lymphadenitis [3]. In addition to a causative agent in genital ulcers, nongenital skin infections with *H*. *ducreyi* are increasingly recognised as a cause of cutaneous ulcers [4]. Point prevalence studies in Papua New Guinea, the Solomon Islands, Vanuatu, and Ghana—all yaws-endemic countries—demonstrated that *H*. *ducreyi* is a leading cause of cutaneous ulcers and even outnumbers yaws [4].

Skin infections caused by *H*. *ducreyi* are uncommon in travellers and have been described in 4 case reports, but may be an underreported or underdiagnosed cause of extragenital ulcers in this group [5, 6]. All patients acquired the infection on one of the Pacific Islands. A report from New Zealand presented 3 cases of lower-limb ulceration caused by *H*. *ducreyi* in children who had visited Samoa [7]. Another paper reported a case of HD-CU in a female crew member from Vanuatu who worked on a visiting cruise ship and presented to an Australian hospital [8]. A third publication reported 2 cases of cutaneous infection imported by Australian expatriates from Papua New Guinea and Vanuatu, respectively [9].

Like in the present case, a 22-year-old Danish man contracted an HD-CU after a minor trauma in 1987 while swimming near the Fiji islands [10]. A cross-sectional study in Papua New Guinea indeed found high rates of *H*. *ducreyi* DNA on the skin of asymptomatic participants, on flies, and on bed sheets, suggesting that *H*. *ducreyi* survives on healthy, nongenital skin where even minor trauma could initiate infection [11]. In addition, these cases with HD-CU after a minor trauma could indicate that *H*. *ducreyi* may be even more widespread in the environment (e.g., in soil or on rocks) than already established.

In our patient, the infection was very likely contracted in the Maluku province of Indonesia. A literature search revealed no other reports on *H*. *ducreyi* cutaneous ulceration or ‘chancroid’ from Indonesia or the Maluku province. However, the proximity of the Maluku province islands to New Guinea island (Fig 1) suggests that *H*. *ducreyi* is endemic in at least some of the northeastern islands of Indonesia.

The biopsy taken from the margins of the ulcer, which was taken before antibiotics were prescribed, showed a negative *H*. *ducreyi*-specific PCR and bacterial culture and instead showed growth of *C*. *diphtheriae*. This finding could be explained by colonisation of the wound with *C*. *diphtheriae* in combination with a sampling error, causing the negative *H*. *ducreyi* culture and PCR. This is in line with a study examining the location of *H*. *ducreyi* within ulcers that found that bacteria were mainly confined to the ulcer base [12]. However, a polymicrobial infection causing the ulcer cannot be excluded, as *C*. *diphteriae* is also known to cause skin ulceration.

The specific *H*. *ducreyi* PCR has been performed for over 15 years at the PHL in routine diagnostics of genital ulcers [13]. In this timeframe, approximately 600 *H*. *ducreyi* PCRs have been performed without any positives, indicating that *H*. *ducreyi* does not circulate in the Netherlands, rendering the chance that the infection was contracted locally extremely low. Also, specificity of the PCR products in the present case was confirmed by sequencing, supporting the notion that this patient was indeed infected with *H*. *ducreyi*.

Strains of *H*. *ducreyi* causing cutaneous ulcers are genetically highly homologous to genital ulcer strains and have comparable low MICs for azithromycin [14]. Recommended treatment for genital ulcers caused by *H*. *ducreyi* is generally azithromycin or ceftriaxone in a single dose, or ciprofloxacin for 3 days [15]. However, optimal treatment duration for skin ulceration was not established at the time of the present case, and reported cases varied from a single dose to 2-week regimens [8, 9]. Very recently, Camila González-Beiras et al. published results of a community-based cohort study in Papua New Guinea from October 2014 through May 2016 in which they found a single oral dose of azithromycin of 30 mg/kg to be effective for the treatment of HD-CU [16]. Although our patient responded very well, had these results been available, we very likely would have treated with either azithromycin or a shorter course of ciprofloxacin.

This is the second report of a *H*. *ducreyi* strain isolated from a cutaneous ulcer that produces beta-lactamase [17], which warrants monitoring of penicillin resistance in *H*. *ducreyi* isolates, especially since in many tropical countries cutaneous ulcers are treated empirically with penicillin [18].

This case illustrates the role of *H*. *ducreyi* as a possible cause of cutaneous ulcers in travellers and indicates that *H*. *ducreyi* is present in at least some of the northeastern islands of Indonesia.

## Ethics statement

This case is published with permission of the patient, and written informed consent has been obtained.

**Key learning points**

- In addition to a causative agent of genital ulcers, *H*. *ducreyi* is increasingly recognised as a cause of cutaneous ulcers.
- Skin infections caused by *H*. *ducreyi* in travellers are rare and have been described in travellers to the Pacific Islands.
- This case indicates that *H*. *ducreyi* is also present in at least one of the northeastern islands of Indonesia.
- Clinicians should consider *H*. *ducreyi* as a cause of cutaneous ulcers in previously healthy travellers.

## Supporting information

S1 Supplementary material Primer and probe sequences. (DOCX)
