# Supplementary material for: Haemophilus ducreyi cutaneous ulcer contracted at Seram Island, Indonesia, presented in the Netherlands
Source: PLoS Negl Trop Dis. 2018 Apr 12;12(4):e0006273. doi: 10.1371/journal.pntd.0006273 (PMC5896912; doi:10.1371/journal.pntd.0006273)
Supplement: S1 Supplementary material — (DOCX) [file pntd.0006273.s001.docx]

Supplementary material

# Primer and probe sequences

## *Haemophilus ducreyi*

The primers for *H. ducreyi* used in a nested PCR format are located in the *16S rRNA* gene.

Outer primers, with a fragment length of 960 bp, were

Forward outer primer: HD07: 5’ CAA GTC GAA CGG TAG CAC GAA G and

Reverse outer primer: HD roe2: 5’ TCA TCT CTG AGT TCT TCT ATG

Inner primers, with a fragment length of 309 bp, were

Forward inner primer : HD08: 5’ TTC TGT GAC TAA CGT CAA TCA ATT TTG and

Reverse inner primer: HD14: 5’ TCG GAT TAA AGG GTG GGA CCT T

These were partly published in:

- Orle KA, Gates CA, Martin DH, Body BA, and Weiss JB.. Simultaneous PCR detection of Haemophilus ducreyi, Treponema pallidum, and herpes simplex virus types 1 and 2 from genital ulcers. J. Clin. Microbiol. 1996;34:49–54.

- Roesel DJ, Gwanzura L, Mason PR, Joffe M, and Katzenstein DA.. Polymerase chain reaction detection of Haemophilus ducreyi DNA .Sex. Transm. Infect. 1998;74:63–65.

and in detail in:

- Bruisten SM, Cairo I, Fennema H, Pijl A et al. Diagnosing genital ulcer disease in a clinic for sexually transmitted diseases in Amsterdam, The Netherlands. J Clin Microbiol. 2001 Feb;39(2):601-5.

## Treponema pallidum

Detection of the *PolA* gene with a real time PCR for *T. pallidum* subspecies:

Forward primer: TPs: 5'- GGT AGA AGG GAG GGC TAG TA-3'

Reverse primer: TPas 5'- CTA AGA TCT CTA TTT TCT ATA GGT ATG G-'3

Fragment length: 104 bp

Probe: TP-TQ 5'-FAM ACA CAG CAC TCG TCT TCA ACT CC-BHQ1-3'

Published in:

- Koek AG, Bruisten SM, Dierdorp M, van Dam AP, Templeton K. Specific and sensitive diagnosis of - syphilis using a real-time PCR for Treponema pallidum. Clin Microbiol & Infect. 2006;12:1233-1236.

- Heymans R, van der Helm JJ, de Vries HJC, Fennema HAS, Coutinho RA, Bruisten SM Clinical value of Treponema pallidum real-time PCR for diagnosis of syphilis. J Clin Microbiol. 2010;48(2):497-502.

## *Mycobacterium tuberculosis* complex

Detection of the insertion sequence IS6110, an insertion element found exclusively within the members of the *Mycobacterium tuberculosis* complex (MTBC)

| Name | Sequence | Bases (n) |
| --- | --- | --- |
| MtubF2 | CCG CTT CGG ACC ACC A | 16 |
| MtubR2 | GGT GAC AAA GGC CAC GTA GG | 20 |
| MtubP2 | 6-FAM- CCG GCT GTG GGT AGC –MGBNFQ | 15 |
